# Embolic strokes of undetermined source in a cohort of Polish stroke patients

**DOI:** 10.1007/s10072-018-3322-5

**Published:** 2018-03-19

**Authors:** Jan Pawel Bembenek, Michal Adam Karlinski, Iwona Kurkowska-Jastrzebska, Anna Czlonkowska

**Affiliations:** 1 2nd Department of Neurology, Institute of Psychiatry and Neurology, ul. Sobieskiego 9, 02-957 Warsaw, Poland; 2 Department of Experimental and Clinical Pharmacology, Medical University of Warsaw, Warsaw, Poland

**Keywords:** Stroke classification, Embolic stroke of undetermined source, Ischemic stroke, Embolism, Cryptogenic stroke

## Abstract

We aimed to provide a descriptive analysis of embolic stroke of undetermined etiology (ESUS) population based on a long-term prospective stroke registry. We retrospectively analyzed data collected in a detailed registry regarding consecutive patients admitted for first-ever ischemic stroke (IS) between January 2001 and December 2015. We used Org 10172 in Acute Stroke Treatment classification supplemented with ESUS criteria proposed by the Cryptogenic Stroke/ESUS International Working Group. Within the ESUS group, we additionally compared patients ≤ 60 and > 60 years of age. During the study period, there was a total of 3008 (1615 females and 1393 males) admissions of first-ever strokes. The most frequent cause was undetermined (38.7%), followed by cardioembolic (27.7%), large artery atherosclerosis (18.2%), small vessel disease (11.9%), and other determined (3.6%). We identified 326 patients as ESUS, which accounted for 10.8% of all strokes and 28% of strokes of undetermined etiology. ESUS patients were the youngest. Compared to all types of stroke but for those with small vessel disease, ESUS patients were most often independent before stroke and had the least severe neurological deficit at admission and the best outcome at discharge. ESUS patients ≤ 60 years were more frequently independent at discharge than ESUS patients > 60 years. Approximately 11% of patients from our registry met ESUS criteria. ESUS patients were younger when compared to all other stroke etiologies, suffered less severe strokes, and had more favorable outcome at discharge than other groups except for those with small vessel disease strokes.

## Introduction

In the frequently used Trial of ORG 10172 in Acute Stroke Treatment (TOAST) classification system, stroke of undetermined cause may refer indeed to a stroke with no identified etiology after investigations are complete but also to a stroke with incomplete diagnostic workup or to stroke with more than one possible cause [1]. A new clinical entity termed embolic stroke of undetermined source (ESUS) was recently introduced by the Cryptogenic Stroke/ESUS International Working Group [2]. It describes a stroke that is likely to be of embolic origin but the source of the embolism remains undetected despite completed standard investigation. The list of potential etiologies underlying ESUS includes minor-risk potential cardioembolic sources, covert paroxysmal atrial fibrillation, cancer-associated coagulopathy and embolism, arteriogenic emboli and paroxysmal embolism, in situ thrombosis, prothrombotic disorders, and others [2].

From a therapeutic viewpoint, it is important to differentiate the underlying causes of stroke. Patients with cryptogenic embolic stroke show distinct clinical and radiological features depending on the underlying causes, such as aortic arch atheroma, patent foramen ovale, or paroxysmal atrial fibrillation [3]. Moreover, patients classified as ESUS may differ from cryptogenic strokes and other groups classified according to TOAST criteria in terms of age (younger), stroke severity (mild), outcome (better), and required secondary stroke prevention (oral anticoagulants) [4, 5]. There is still need for high quality data regarding frequency and clinical features of ESUS patients, when compared to other stroke etiologies.

We aimed to provide a detailed descriptive analysis of a real-life ESUS population derived from a prospective long-term stroke registry in Polish urban setting using the proposed diagnostic criteria and to compare it with other stroke etiologies.

## Methods

We analyzed consecutive acute stroke patients admitted between January 2001 and December 2015 to a single stroke center providing neurological care for a population of approximately 200,000–250,000 inhabitants of a highly urbanized area (Warsaw, Poland). Data were prospectively collected in a detailed stroke registry developed as an adaptation of the National Institute of Neurological and Communicative Disorders and Stroke Data Bank protocols, which was described in details elsewhere [6, 7]. The collected information included patients’ demographics, risk factors and comorbidities, prestroke medications, results of routine laboratory tests, and status at discharge.

The diagnosis of acute stroke was based on World Health Organization (WHO) definition [8]. Routine brain imaging at admission included non-contrast brain computed tomography (CT), which in selected cases was complemented or substituted with magnetic resonance imaging (MRI). Neurological deficit was measured with National Institutes of Health Stroke Scale (NIHSS) [9]. Most diagnostic procedures necessary to diagnose ESUS patients were provided routinely in our stroke unit. All patients underwent at least 24-h electrocardiographic (ECG) telemetry, which in many cases was supplemented with Holter-type 24-h ECG monitoring and transthoracic echocardiography (TTE). TTE was not provided in all patients, so we classified them as potential ESUS: patients fulfilling all other ESUS criteria but without performed TTE. Ischemic stroke subtypes (large artery atherosclerosis, small vessel disease, cardioembolic, undetermined, and other determined) were classified according to modified TOAST criteria [2]. Patients with more than one probable cause of stroke classified according to original TOAST are classified as having stroke of unknown etiology. In our study, this group was split into “truly” unknown etiology and mixed etiology. The outcome was evaluated in the modified Rankin Scale (mRS) score at discharge from the stroke unit [10]. Unfavorable outcome was defined as death or dependency (mRS score 3–6).

Patients classified as ESUS were extracted from the group with undetermined stroke according to TOAST classification. ESUS was defined as proposed by the Cryptogenic Stroke/ESUS International Working Group [2] according to data available in our registry. Detailed classification of stroke etiologies is given in Table 1.

**Table 1:** Types of ischemic stroke in a cohort of 3008 first-ever stroke patients

Type of ischemic stroke	Number	Percent
Undetermined strokes	1163	38.7
ESUS*	326	10.8
Potential ESUS**	288	9.6
Unknown	461	15.3
Mixed pathology	88	2.9
Large artery atherosclerosis	547	18.2
Cardioembolic***	833	27.7
Small vessel disease	358	11.9
Other determined	107	3.6

*ESUS, embolic stroke of undetermined source: undetermined according to TOAST and confirmed no atrial fibrillation (AF), no unilateral stenosis ≥ 50%, and no source of embolism on transthoracic echocardiography (TEE) and confirmed ischemic lesion in neuroimaging at admission or in follow-up brain imaging

**Patients fulfilling all other ESUS criteria but without performed TTE

***Fraction of cardioembolic due to non-valvular AF was 95%

The registry was developed in concordance with the Declaration of Helsinki and was approved by the local ethics committee.

### Statistical methods

Categorical variables were presented as a number of valid observations and proportions calculated with exclusion of unknown values from the denominator. Due to non-normal distribution, continuous variables were presented as a median with interquartile range (first quartile to third quartile, Q1–Q3).

Comparisons between particular groups of patients were done using the chi-square test or Kruskal-Wallis test, as appropriate. If the overall test for significance was positive (*p* < 0.05), pairwise comparisons were made between the ESUS group and each other group. Such approach allowed to reduce the risk of type I error without losing power by applying the Bonferroni correction.

*p* values of < 0.05 were considered statistically significant. Calculations were carried out using STATISTICA 12.0 software package (Stat Soft Inc., Tulsa, USA, 2013).

## Results

Study population consisted of 3008 consecutive patients (1615 females and 1393 males) admitted due to the first-ever acute ischemic stroke. The characteristic of the studied cohort according to stroke subtype is presented in Table 1.

A total of 1163 (38.7% of all strokes) patients were classified as having stroke of undetermined etiology, of whom 326 (10.8%) were subsequently recognized as definite ESUS and 288 (9.6%) due to not completed diagnostic workup as potential ESUS.

ESUS patients were the youngest (median age 64.5 vs 67–79 in other groups; *p* < 0.05) and had less severe neurological deficit (median NIHSS score 5; *p* < 0.05) when compared to all other groups (median NIHSS score 8–16; *p* < 0.05) except for patients with strokes caused by small vessel disease (median NIHSS score 4; *p* < 0.05). They were also more often independent before stroke (mRS 0–1) compared to other groups (90.5 vs 67.1–80.2%; *p* < 0.05) except for small vessel disease (86.3%; *p* > 0.05). Only patients with large artery atherosclerosis were smoking more frequently than ESUS patients (47.3 vs 39.6%; *p* < 0.05) while patients from other groups were less often current smokers (14.8–30.8%; *p* < 0.05) (Table 2).

**Table 2:** Baseline characteristic and outcome at discharge from the hospital according to ischemic stroke etiology

	ESUS *n* = 326	Large artery atherosclerosis [a] *n* = 547	Cardioembolic [b] *n* = 833	Small vessel disease [c] *n* = 358	Other determined [d] *n* = 107	Potential ESUS (diagnostics not completed) [e] *n* = 288	Mixed pathology [f] *n* = 88	Unknown [g] *n* = 461	Overall *p*	Pairwise differences vs ESUS
At admission										
Age, median (Q1, Q3)	64.5 (57, 72)	70 (62, 77)	79 (72, 84)	71 (60, 70)	67 (55, 78)	79 (72, 83)	79 (74, 85)	75 (65, 81)	< 0.001	a, b, c, e, f, g
Females, *n* (%)	150 (46.0)	209 (38.2)	529 (63.5)	183 (51.1)	58 (54.2)	166 (57.6)	58 (65.9)	262 (56.8)	< 0.001	a, b, e, f, g
NIHSS, median (Q1, Q3)	5 (3, 10)	8 (4–15)	11 (5–18)	4 (2, 7)	8 (3, 16)	8 (4, 14)	16 (6–22)	6 (3, 12.5)	< 0.001	a, b, c, d, e, f
Prestroke mRS 0–1, *n* (%)	295 (90.5)	436 (80.2)	567 (68.2)	308 (86.3)	84 (78.5)	205 (71.2)	59 (67.1)	346 (75.6)	< 0.001	a, b, d, e, f, g
Hypertension, *n* (%)	233 (71.7)	419 (77.2)	666 (80.4)	293 (81.8)	66 (62.3)	220 (77.2)	75 (85.2)	313 (68.3)	< 0.001	b, c, f
Atrial fibrillation, *n* (%)	–	–	–	–	3 (2.8)	–	82 (93.2)	–	< 0.001	
Coronary artery disease, *n* (%)	67 (20.7)	148 (27.4)	332 (41.3)	83 (23.5)	16 (15.1)	80 (28.4)	40 (47.6)	134 (29.7)	< 0.001	a, b, e, f, g
Heart failure, *n* (%)	31 (9.6)	70 (12.9)	305 (37.8)	27 (7.6)	12 (11.3)	41 (14.5)	36 (42.4)	82 (18.1)	< 0.001	b, f, g
Diabetes, *n* (%)	59 (18.1)	133 (24.3)	202 (24.3)	86 (24.0)	17 (15.9)	61 (21.2)	25 (28.4)	86 (18.7)	0.034	a, b, f
Current smoking, *n* (%)	128 (39.6)	256 (47.3)	122 (14.8)	109 (30.8)	30 (28.0)	58 (20.5)	17 (20.0)	100 (21.8)	< 0.001	a, b, c, d, e, f, g
Hospital stay										
Hospital stay (days), median (Q1, Q3)	10 (8, 14)	11 (8–15)	12 (9–18)	9 (7, 11)	10 (8–16)	10 (8, 15)	11 (7–18)	9 (7, 13)	< 0.001	b, c
Holter ECG, *n* (%)	263 (81.2)	325 (59.6)	425 (51.2)	217 (60.6)	57 (53.8)	126 (43.9)	33 (37.5)	268 (59.3)	< 0.001	a, b, c, d, e, f, g
TTE, *n* (%)	326 (100)	393 (78.3)	555 (67.8)	269 (99.3)	78 (100)	109 (37.9)	47 (69.1)	342 (100)	< 0.001	
At discharge										
Anticoagulants at discharge, *n* (%)	27 (8.3)	53 (9.7)	341 (40.9)	12 (3.4)	16 (15.0)	26 (9.0)	31 (35.2)	25 (5.4)	< 0.001	b, c, d, f
mRS 0–2 at discharge, *n* (%)	213 (65.5)	246 (45.1)	297 (36.0)	274 (76.8)	47 (43.9)	118 (41.4)	27 (31.0)	268 (58.5)	< 0.001	a, b, c, d, e, f, g
Death during hospital stay, *n* (%)	7 (2.2)	54 (9.9)	112 (13.5)	6 (1.7)	12 (11.2)	38 (13.2)	26 (29.6)	50 (10.9)	< 0.001	a, b, d, e, f, g

*ESUS* embolic stroke of undetermined source, *NIHSS* National Institutes of Health Stroke Scale, *mRS* modified Rankin Scale, *AF* atrial fibrillation, *ECG* electrocardiography, *TTE* transthoracic echocardiogram

Patients classified as ESUS had significantly more often favorable outcome (mRS 0–2) at discharge from the hospital compared to all other subtypes of stroke (65.5 vs 31.0–58.5%; *p* < 0.05) but for small vessel disease (76.8%; *p* < 0.05). Death during hospital stay was also significantly less common in ESUS patients when compared to other groups (2.2 vs 9.9–29.6%; *p* < 0.05) with exception of small vessel disease (1.7%; *p* > 0.05) (Table 2). Oral anticoagulants were prescribed at discharge to 8.3% of ESUS patients.

Patients < 60 years of age constituted 32.2% of the ESUS group. They were more frequently independent before stroke (mRS 0–1), more often current smokers, but less often burdened with coronary artery disease or heart failure compared to patients ≥ 60 years of age. Younger ESUS patients had more frequently good outcome (mRS 0–2) at discharge (76 vs 60.6%; *p* = 0.007) but with no significant difference in terms of mortality (Table 3).

**Table 3:** Comparison characteristic of ESUS patients < 60 and ≥ 60 years of age

	ESUS aged < 60 *n* = 105	ESUS aged ≥ 60 *n* = 221	*p* value
Age, median (Q1, Q3)	52 (47, 56)	69 (64, 75)	< 0.001
Females, *n* (%)	43 (41.0)	107 (48.4)	0.206
Prestroke mRS 0–1, *n* (%)	102 (97.1)	193 (87.3)	0.004
Hypertension, *n* (%)	69 (66.4)	164 (74.2)	0.142
Coronary artery disease, *n* (%)	8 (7.6)	59 (26.9)	< 0.001
Heart failure, *n* (%)	2 (1.9)	29 (13.2)	0.001
Diabetes, *n* (%)	11 (10.5)	48 (21.7)	0.014
Current smoking, *n* (%)	66 (62.9)	62 (28.4)	< 0.001
NIHSS, median (Q1, Q3)	5 (3, 10)	5 (3, 10)	0.748
Hospital stay (days), median (Q1, Q3)	10 (8, 14)	10 (8, 14)	0.852
Holter ECG, *n* (%)	85 (81.7)	178 (80.9)	0.860
mRS 0–2 at discharge, *n* (%)	79 (76.0)	134 (60.6)	0.007
Death at discharge, *n* (%)	1 (1.0)	6 (2.7)	0.341
Anticoagulants at discharge, *n* (%)	7 (6.7)	20 (9.1)	0.466

*ESUS* embolic stroke of undetermined source, *NIHSS* National Institutes of Health Stroke Scale, *mRS* modified Rankin Scale, *AF* atrial fibrillation, *ECG* electrocardiography

## Discussion

ESUS have been considered potentially more responsive to oral anticoagulation than antiplatelet therapy for secondary stroke prevention [2, 11–13]. Ongoing trials aim to evaluate the efficacy and safety of novel oral anticoagulants versus acetylsalicylic acid in ESUS patients and their results are eagerly awaited [12–14]. It is highly probable that the origin of stroke is cardioembolic; hence, a currently published study suggests that prolonged monitoring could improve the detection of atrial fibrillation in ESUS patients [15]. Moreover, it has been recently demonstrated that ECG parameters may be helpful in detection of cardioembolic origin of stroke [16, 17]. Other authors suggest that atrial biomarkers (left atrial diameter on echocardiography, P-wave terminal force in ECG lead V1, and P-wave-R-wave (PR) interval on ECG) are weakly associated with atrial fibrillation after ESUS [18]. However, according to the current review of the published studies, most (86%) ESUS patients were treated with antiplatelet therapy during follow-up [11]. Hence, it is important to identify such patients and provide them proper secondary prevention. However, descriptive analysis and comparison with strokes of other origin is also important as the amount of data from large cohorts of patients is still not sufficient [5, 19]. The strength of our study is that it is a population-based with large cohort of non-selected consecutive first-ever stroke patients and almost complete baseline characteristic data.

Previously two large cohort studies [5, 19] aimed to describe ESUS population using the criteria proposed by the Cryptogenic Stroke/ESUS International Working Group [2]. Other small studies included only 100–236 patients [15, 20, 21]. Systematic review of the literature identified nine published studies evaluating ESUS patients [11]. Moreover, there is still little data regarding stroke outcome when compared to other stroke etiologies.

Approximately one-third of all ischemic strokes are of undetermined etiology according to TOAST classification and they are more prevalent among young adults [22–25] and teens and young adults < 40 years [26]. ESUS strokes are estimated as 9–33% of all acute ischemic strokes, reaching over 40% in some small series [20, 21]. In our cohort, ESUS criteria were met by approximately 11% of first-ever ischemic stroke patients. Another 10% of patients were potentially ESUS, but did not complete diagnostic procedures as TTE.

Patients with ESUS had less severe neurological deficit at admission (median NIHSS score 5), when compared to other stroke types (median NIHSS 6–21), and only the small vessel disease group had lower NIHSS score (median 4). Additionally, ESUS patients were more independent in daily activities before stroke and younger. Neurological deficit of moderate severity when compared to other stroke etiologies was also reported in ESUS patients by Ntaios et al. (NIHSS score 5 [2–14]) [19] and Martinez-Majander et al. (NIHSS score 2 [1–6]) [26] but not by Ladeira et al. (mean NIHSS score 4) [20]. Our data are consistent with systematic review of published studies evaluating ESUS patients, where frequency of ESUS ranged from 9 to 25% of all ischemic strokes, the mean age was 65 years, 42% were women, and the mean NIHSS score was 5 at stroke onset [11]. Some discrepancies between studies may result from differences in methodology and frequency in providing diagnostic workup such as Holter ECG and TTE in various countries.

The prediction of outcome following stroke is a challenge. Many attempts have been undertaken to determine the most predictive variables including clinical, laboratory, pharmacological, and radiologic fields [27–31]. Major findings of the current study were the association of ESUS with better outcome compared to other stroke etiologies and the greater likelihood of good recovery among younger than older ESUS patients. In our cohort, ESUS patients had better outcome at discharge, as almost 66% of patients had mRS 0–2, when compared to 31–59% in other stroke etiologies, except for small vessel disease patients in whom the proportion of mRS 0–2 was higher (approx. 77%). The risk of death during hospital stay was also lower in the ESUS group (approx. 2%), when compared to other groups (approx. 10–30%). This is consistent with other results [4, 25]. Arauz and colleagues [25] and Ntaios et al. [4] reported more favorable outcome in ESUS patients (60.4 and 62.5%, respectively). Moreover, in Arauz et al. study, the ESUS group had lower risk of death [25].

ESUS patients younger than 60 years may differ in terms of vascular risk factors, stroke etiology, severity, and outcome when compared to older patients. Data from the recently published ESUS Global Registry have shown a substantial percentage (about 20%) of ESUS patients to be under the age 50 with high fractions from Latin American and East Asian countries [5], but there is little data regarding European ESUS patients younger than 60 years [20, 26]. Besides, the study of Ladeira et al. is limited by small sample size [20] and Martinez-Majander et al. reported only patients aged 15 to 40 years [26]. Hence, there has been a gap in the knowledge of younger than 60 years ESUS patients. Noteworthy, patients < 60 years of age accounted for 32% of the entire ESUS group. Considering their young age, they may require more detailed diagnostic workup. In our study, ESUS patients < 60 years substantially differed from older patients and were more frequently independent before stroke and more frequently current smokers but less frequently burdened with coronary artery disease and heart failure when compared to patients ≥ 60 years of age. There was no significant difference in the mortality rate during hospital stay between those groups but patients < 60 years more frequently had no prestroke disability (mRS ≤ 1) and were less burdened with vascular risk factors. Ntaios et al. [32] recently reported an over fourfold increase of the risk of all-cause mortality in ESUS patients aged 60 to 80 years and an eightfold increase in those aged > 80 years compared to those < 60 years of age during the median follow-up of 31 months.

Introduction of TOAST classification made it easier to standardize the stroke patients according to the stroke origin and encouraged more detailed diagnostic workup which resulted in optimized secondary stroke prevention. However, according to TOAST, there is a risk of classification of patients with various coexisting risk factors in one group. Hence, we modified TOAST classification for the purposes of our study. ESUS is a relatively novel clinical construct [2]. It seems to be more accurate for stroke patients than TOAST classification. Further, well-designed prospective studies could inform us about functional outcome of ESUS patients, stroke recurrence, and overall and cardiovascular mortality.

Our study has certain limitation. It is a retrospective analysis of stroke patients with first-ever ischemic stroke admitted to a stroke center in a highly urbanized area, which may not be representative to whole country. It is probable that younger stroke patients more often underwent extensive diagnostic workup required to meet ESUS criteria, while patients with more severe strokes and/or early in-hospital death did not complete the workup. This could have biased the results. For that reason, we distinguished group with potential ESUS. Therefore, the actual proportion of ESUS patients is probably higher than 10.8% but definitely lower than 20.4%. On the other hand, the fact that they differed in many aspects from the ESUS group suggests that potential ESUS were not typical ESUS patients.

## Conclusion

Approximately 11% of patients with first-ever ischemic stroke met criteria for ESUS. Patients with ESUS were younger and had less severe strokes and better outcome compared to other stroke types, but small vessel disease. Younger ESUS patients had more frequently good outcome at discharge but with no significant difference in terms of mortality. We believe that these results add to the pool of knowledge about this group of patients, especially originating from Central and Eastern Europe. This may be useful in both clinical and research setting.
